# Benchmarking UMI-aware and standard variant callers for low frequency ctDNA variant detection

**DOI:** 10.1186/s12864-024-10737-w

**Published:** 2024-09-03

**Authors:** Rugare Maruzani, Liam Brierley, Andrea Jorgensen, Anna Fowler

**Affiliations:** 1 Department of Health Data Science, Institute of Population Health, University of Liverpool, Waterhouse Building, Block F, Brownlow Street, Liverpool, L69 3GF UK; 2 MRC-University of Glasgow Centre for Virus Research, University of Glasgow, Garscube Campus, 464 Bearsden Road, Glasgow, G61 1QH UK

**Keywords:** Variant calling, ctDNA, Next generation sequencing, Cancer

## Abstract

**Background:**

Circulating tumour DNA (ctDNA) is a subset of cell free DNA (cfDNA) released by tumour cells into the bloodstream. Circulating tumour DNA has shown great potential as a biomarker to inform treatment in cancer patients. Collecting ctDNA is minimally invasive and reflects the entire genetic makeup of a patient’s cancer. ctDNA variants in NGS data can be difficult to distinguish from sequencing and PCR artefacts due to low abundance, particularly in the early stages of cancer. Unique Molecular Identifiers (UMIs) are short sequences ligated to the sequencing library before amplification. These sequences are useful for filtering out low frequency artefacts. The utility of ctDNA as a cancer biomarker depends on accurate detection of cancer variants.

**Results:**

In this study, we benchmarked six variant calling tools, including two UMI-aware callers for their ability to call ctDNA variants. The standard variant callers tested included Mutect2, bcftools, LoFreq and FreeBayes. The UMI-aware variant callers benchmarked were UMI-VarCal and UMIErrorCorrect. We used both datasets with known variants spiked in at low frequencies, and datasets containing ctDNA, and generated synthetic UMI sequences for these datasets. Variant callers displayed different preferences for sensitivity and specificity. Mutect2 showed high sensitivity, while returning more privately called variants than any other caller in data without synthetic UMIs – an indicator of false positive variant discovery. In data encoded with synthetic UMIs, UMI-VarCal detected fewer putative false positive variants than all other callers in synthetic datasets. Mutect2 showed a balance between high sensitivity and specificity in data encoded with synthetic UMIs.

**Conclusions:**

Our results indicate UMI-aware variant callers have potential to improve sensitivity and specificity in calling low frequency ctDNA variants over standard variant calling tools. There is a growing need for further development of UMI-aware variant calling tools if effective early detection methods for cancer using ctDNA samples are to be realised.

**Supplementary Information:**

The online version contains supplementary material available at 10.1186/s12864-024-10737-w.

## Background

Cancer is one of the leading causes of mortality worldwide. Late diagnosis of cancer is associated with increased mortality and morbidity [1], therefore early screening methods are crucial in improving cancer outcomes. For some cancer types, screening can be invasive while other types have ineffective or no screening tests. This ultimately leads to low screening compliance for invasive tests, and detection of cancer in late stages of disease where screening tests are subpar [2].

Tissue biopsy, the gold standard in cancer diagnosis and monitoring, is associated with a range of challenges that ultimately hinder early diagnosis and monitoring of disease progression [3]. For diagnosis, tumours may be inaccessible for biopsy [4]. Tissue biopsies are also invasive and can cause distress to patients. This also impairs disease monitoring as repeated tissue sampling is not generally feasible.

Further, the utility of tissue biopsy for personalised medicine is limited by the fact that tumours can display temporal and spatial intratumoral heterogeneity. That is, mutations detected in a tumour biopsy may differ in distal subclones in the case of metastasis, or in different sites within the same mass for localised tumours [4].

All tissues release fragmented DNA into the bloodstream, collectively called cell free DNA (cfDNA). In cancer patients, circulating tumour DNA (ctDNA) is the subset of cfDNA originating from tumour tissues [5]. Circulating tumour DNA has shown potential as a minimally invasive biomarker for early diagnosis, personalised treatment, and disease monitoring in cancer patients [6–8]. Collecting ctDNA is minimally invasive as only a blood sample is required. Additionally, ctDNA is released into the bloodstream in the early stages of cancers which may enable diagnosis at earlier stages of disease than other methods [9]. Tracking of ctDNA mutations can also inform prognosis and predict response to treatment [10].

While other approaches like methylation analysis [11] and digital droplet PCR [12] are available, detection of cancer variants in NGS data is often the preferred method for ctDNA analysis. Variants detected in NGS data can indicate the development of resistance to therapeutics [13], predict risk of relapse [14], and inform personalised treatment options. The accurate detection of ctDNA variants in next-generation sequencing data (NGS) is therefore critical in realising the potential of ctDNA analysis as a minimally invasive cancer biomarker.

Circulating tumour DNA variants, however, can present a challenge for variant calling tools due to the low variant allele frequencies expected in ctDNA NGS data. While some tumours release more cfDNA than others [15], generally, the abundance of ctDNA in the bloodstream is correlated with the size of the tumour mass where it originated [16]. Ultimately, ctDNA variants in NGS data may occur at frequencies difficult to distinguish from NGS error rates, especially in the early stages of disease where tumours are comparatively smaller, or in the presence of subclones [17].

The PCR step of most NGS workflows introduces low frequency artefacts which can be misidentified as low frequency variants. Unique Molecular Identifiers (UMIs) can help reduce the number of NGS artefacts during data analysis. UMIs are short random nucleotide sequences tagged to DNA fragments before amplification. Variants observed in individual reads within a UMI family can be assumed to be PCR or sequencing artefacts and filtered out. True positive variants, on the other hand, will be represented in all PCR duplicate reads within the same UMI family. In this way, UMIs can increase the confidence of called variants at the low allele frequencies expected in ctDNA NGS data [18]. Early cycle PCR artefacts can complicate artefact filtering with UMIs as errors are propagated in later copies and become difficult to distinguish from true variants [19].

Fgbio [20] is one of the widely used toolkits for processing UMI encoded NGS data before variant calling. A typical workflow for processing UMI barcoded NGS data with the fgbio toolkit involves multiple steps to annotate binary alignment map (BAM) files with UMI sequences and generate molecular consensus reads.

While the majority of available variant callers require this multistep workflow before calling variants on UMI encoded data, there are a number of variant callers that natively process UMI sequences [21–23]. These UMI-aware variant callers employ custom algorithms for generating consensus reads and variant calling.

Benchmarking variant callers is routine when new tools are published [17, 24], however, no peer-reviewed studies independently benchmark tools on ctDNA data. In this study we evaluated the performance of six variant callers, including two UMI-aware tools, for detection of variants in ctDNA datasets with and without synthetic UMI sequences. The variant callers tested were selected based on popularity gauged by number of citations, ability to call low frequency variants, and ability to natively process UMI sequences. We made use of synthetic datasets as they allow user-specified variants to be spiked-in at desired allele frequencies and depths enabling us to accurately assess performance. We also benchmarked tools on publicly available ctDNA samples from metastatic breast cancer (mBC) patients. Synthetic UMIs were generated for both the dataset with spiked-in variants and the mBC dataset to enable a comparison between UMI-aware callers and standard callers combined with UMI-correction methods.

## Methods

### Generating FASTQ files with spiked-in variants

To benchmark the performance of variant callers at calling low frequency variants in ctDNA datasets, we used a set of datasets with 303 variants from the Catalogue of Somatic Mutations in Cancer (COSMIC v94) spiked in at 6 different low allele frequencies. The COSMIC variants were randomly selected from a set of variants mapping to target regions in a template cfDNA BAM file. FASTQ files used to generate this template cfDNA BAM file were acquired from the Sequence Read Archive (SRA) with the accession SRR10296599. The sample was from a cfDNA targeted sequencing of a healthy Han Chinese female. The authors used the Roche ctDNA panel kit targeting 17 cancer associated genes [25]. The sequencing library included UMI adapters, however, these were not made available in the data submitted to the SRA. FASTQ files from this sample were mapped to the GRCh38 human reference using BWA-MEM (v0.7.17) [26] with default parameters. The output sequence alignment map (SAM) file was converted to the BAM file format using Samtools (v1.2) [27].

Two modified copies of this BAM were produced – the first containing 303 COSMIC SNP variants (supplementary material) at 100% allele frequency, and the second containing reference alleles at the same loci at 100% frequency. These modified BAM files were input for a custom Python script used to generate paired-end reads, and synthetic UMI FASTQ files with reads containing the 303 COSMIC variants at allele frequencies of approximately 0.005, 0.01, 0.02, 0.04, 0.05, and 0.075.

**Fig. 1:**
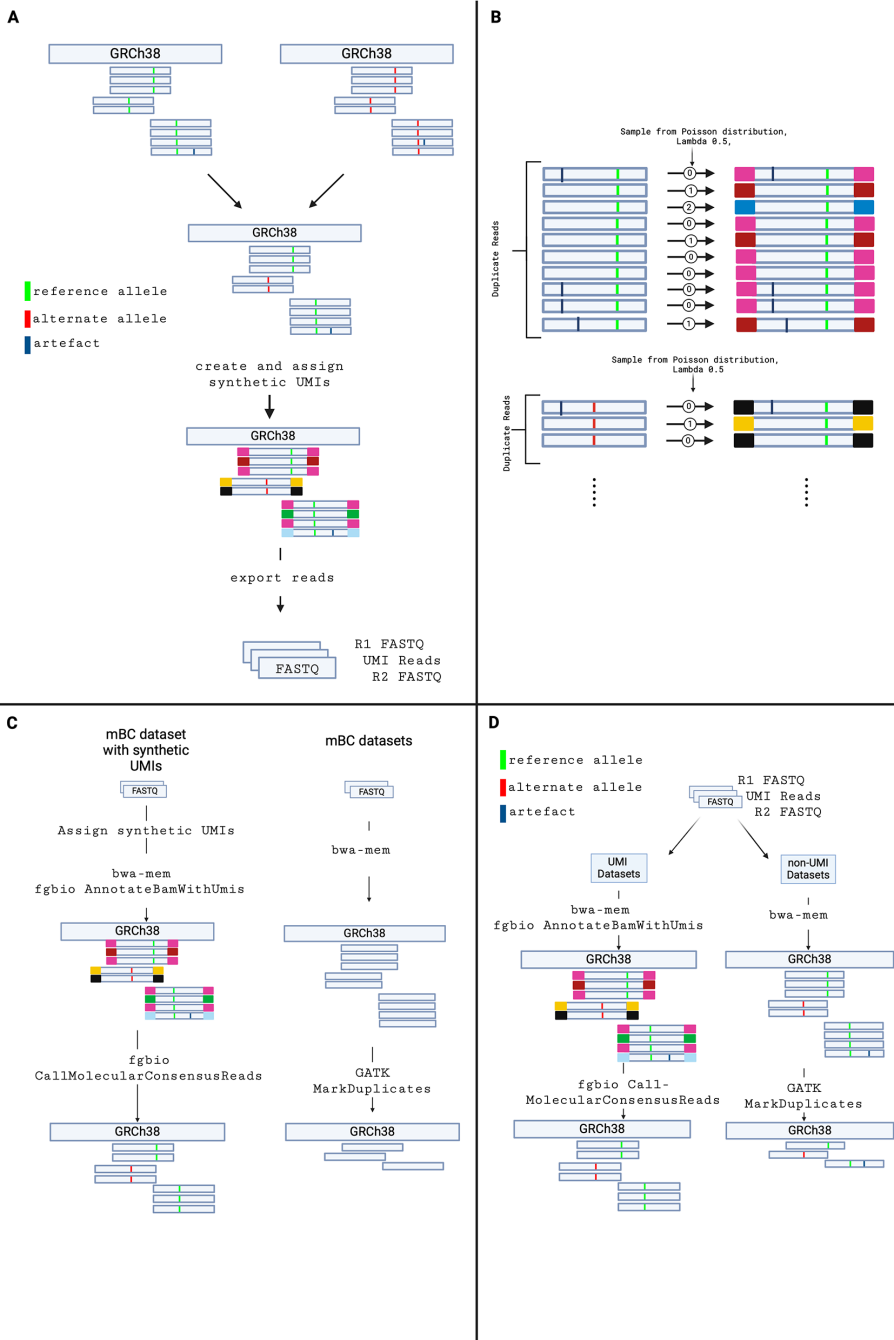
(**A**) Overview of the workflow used to generate FASTQ files with spiked-in variants. A custom Python script was used to assign synthetic UMIs to duplicate reads. For a set of reads with the same start and end positions, each read was assigned a randomly generated synthetic UMI based on sampling from a Poisson distribution. (**B**) Method used to assign synthetic UMIs to duplicate sets of reads. Green bars indicate true variants, black bars indicate artefacts. (**C**) Pre-processing of mBC data with synthetic UMIs and without synthetic UMIs. (**D**) Pre-processing pipeline of datasets with spiked-in variants with and without synthetic UMIs. R1 FASTQ, UMI Reads and R2 FASTQ files are output files from Fig. 1A. To generate UMI encoded data, forward and reverse reads were mapped to GRCh38, and the output BAM file was annotated with UMI sequences. Fgbio was used to call molecular consensus on UMI families. For datasets without synthetic UMIs, forward and reverse reads were mapped to GRCh38, and duplicate reads were removed using the GATK MarkDuplicates tool

Reads from the reference and alternate BAM files were combined to make up desired allele frequencies. The spiked-in data generation process is illustrated in Fig. 1A.

Spiked-in 200x, 450x and 850x datasets at each allele frequency were generated in replicates of 5, at approximately 200x, 450x and 850x depths at the site of spiked-in variants. The spiked-in 200x, dataset excluded ∼0.005 and ∼0.01 allele frequency BAM files due to insufficient read depths meaning that variants at these frequencies often had no reads supporting them.

### Generating synthetic UMIs for the data with spiked-in variants

In the combined BAM file, reads with the same start and stop positions for forward and reverse reads were assumed to be PCR duplicates. Each read within a PCR duplicate set was assigned to UMI families by sampling from a Poisson distribution where the lambda parameter was equal to 1/2. With this method, all reads within a UMI family shared the same start and end position, but not all reads with the same start and end positions share the same UMI sequences. Groups of duplicate reads were preserved when combining reads from the reference and alternate BAM files, to ensure spiked-in variants were assigned to the same UMI family during UMI assignment. Figure 1B illustrates the method of assigning synthetic UMIs to duplicate reads.

UMI sequences were created by generating a random string of letters (A, T,C or G) for the length of the UMI sequence, which was set to 9. The PHRED qualities were set to 37 for all UMI bases.

### Description of metastatic breast cancer (mBC) samples

The publicly available ctDNA samples originated from a 2021 study [28] utilising samples collected from the COMET clinical trial (Clinical Trials Identifier: NCT01745757, Registration Date: 10 Dec 2012). The study included collection of ctDNA from 198 metastatic breast cancer patients at two timepoints – prior to, and four weeks post-treatment. Library construction used a custom panel targeting 54 genes. The sequencing library also included UMI adapters which were not available for download from the SRA. We randomly selected 8 pre-treatment samples from this dataset for benchmarking (SRR15081468, SRR15081470, SRR15081472, SRR15081477, SRR15081480, SRR15081482, SRR15081494, SRR15081493). Raw FASTQ files were downloaded from the SRA using corresponding accession numbers.

### Generating synthetic UMIs for mBC samples

Since the UMIs for the mBC samples were unavailable, synthetic UMIs were generated and artificially added to the samples. Groups of reads with the same start and stop points were assumed to be duplicate reads and assigned to UMI families via random draws from a Poisson process, as illustrated in Fig. 1B.

### Pre-processing data

To generate BAM files in both spiked-in and mBC datasets, reads were aligned to GRCh38 with BWA-MEM at default parameters. Output SAM files were converted to BAM file format and indexed using Samtools (v1.2).

For datasets without synthetic UMIs, read groups were added to output BAM files using the GATK AddOrReplaceReadGroups (v2.27.1) tool, and the GATK MarkDuplicates (v2.27.1) tool was used to mark and discard duplicate reads. The output BAM files were indexed and used as input for variant callers.

In UMI encoded data, fgbio (v1.3.0) [20] was used to annotate BAM files with synthetic UMI sequences and to collapse UMI families into consensus reads. Read groups were added to output BAM files as previously described. These BAM files were input for standard variant callers.

Figure 1C and D illustrate how the mBC dataset and the dataset with spiked-in variants were processed with and without synthetic UMI sequences. Input files for UMI-aware variant callers were either BAM files with synthetic UMIs stored in the RX tag, or FASTQ files with synthetic UMI sequences prepended to read sequences. A custom Python script was used to prepend UMI sequences to FASTQ files.

### Variant calling tools and parameters

The variant callers assessed in this study were bcftools (v1.16) [29], FreeBayes (v1.3.6) [30], LoFreq (v2.1.5) [31], Mutect2 (v4.2.6.1) [32], UMIErrorCorrect (v0.1) [33] and UMI-VarCal (v2.5.0) [34]. While not particularly designed to call low frequency calls, we include bcftools for benchmarking here as one of the most widely used variant callers [35]. Conversely, the authors of LoFreq designed the tool for calling low frequency variants. Several published benchmarking studies have shown Mutect2 and FreeBayes have high sensitivity at calling low frequency variants meriting their inclusion in this study. There is currently only a handful of variant callers that natively support UMI encoded data. UMI-VarCal [34] and UMIErrorCorrect [33] are publicly available, and output the widely used Variant Call Format (VCF) file type to report variants and were therefore included for benchmarking.

All callers were run using default parameters and only variants reported in VCF files were evaluated. UMIErrorCorrect requires a Browser Extensible Data (BED) file which was generated using bedtools (v2.30) [36] and the BAM file from SRR15081472 for the mBC dataset, and SRR10296599 for synthetic datasets. Both UMI-VarCal and UMIErrorCorrect require UMI sequences and were therefore excluded from benchmarking on non-UMI datasets.

### Variant filtering and annotation pipelines

**Fig. 2:**
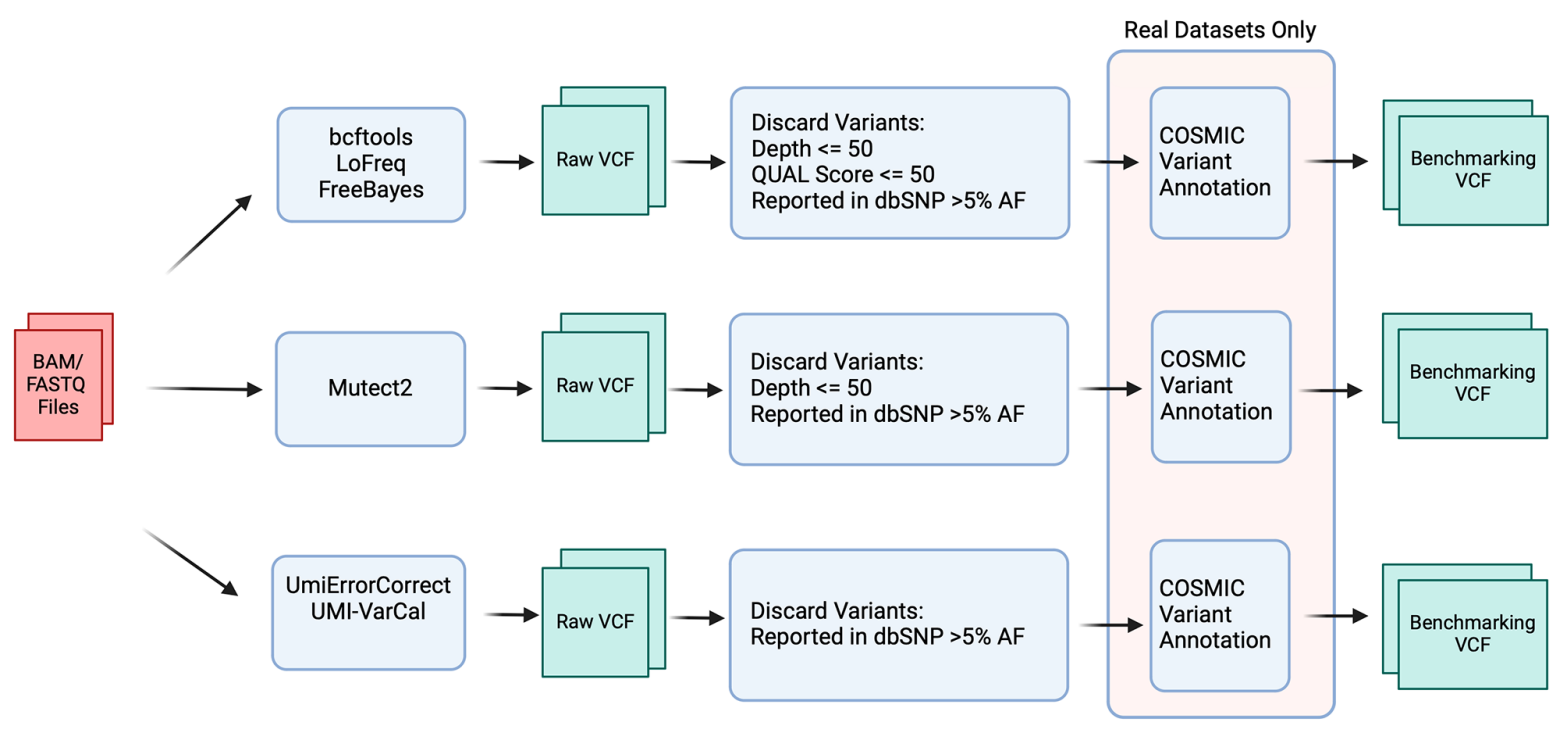
Overview of variant filtering and annotation pipeline

We discarded variants with read depths and per variant quality scores of less than 50. Mutect2 does not report quality scores for called variants, therefore this metric was excluded from variant filtering in Mutect2 VCF files. Next, we used snpSIFT (v4.3t) [37] and bcftools to annotate and discard variants reported in dbSNP v138 at > = 5% in all populations. No depth or base quality score filtering was applied to UMI-aware caller VCF files due to incompatibility with variant filtering tools. To assess performance of callers on mBC datasets, snpSIFT was used to annotate VCF files with COSMIC variants. The COSMIC database VCF file was first filtered to retain variants with sample counts of > = 3. Figure 2 illustrates an overview of the variant filtering pipeline.

### Evaluating variant calls and data visualisation

We assessed the sensitivity of variant callers on the spiked-in datasets using the GATK Concordance tool. The truth set was a VCF file of the 303 spiked-in COSMIC variants. As the data with spiked-in variants originated from a real cfDNA sample, it is difficult to determine if a candidate false postive variant is indeed a false positive, or a real variant in the template data. Therefore, to infer specificity in datasets with spiked-in variants, we calculated the total number of variants detected, minus true positive calls. These variants are refered to as putative false positive variants. In the mBC dataset, the concordance of COSMIC variants called by each caller in sample SRR15081472 was used to gain an insight into false positive discovery rate. The R package UpSetR [38] was used for visualising intersections of called variants.

## Results

Bcftools, FreeBayes, LoFreq and Mutect2 do not support UMI sequences natively. Bcftools, FreeBayes, LoFreq and Mutect2, and UMI-VarCal accept BAM files as input while UMIErrorCorrect accept FASTQ files. UMIErrorCorrect is the only caller to not support indel variant calling (Table 1).

**Table 1:** An overview of benchmarked variant callers

Variant Caller	UMI Awareness	Type of Variant	Input Files
*bcftools*	No	SNP + Indel	BAM
*FreeBayes*	No	SNP + Indel	BAM
*LoFreq*	No	SNP + Indel	BAM
*Mutect2*	No	SNP + Indel	BAM
*UMIErrorCorrect*	Yes	SNP	FASTQ
*UMI-VarCal*	Yes	SNP + indel	BAM

### Overview of benchmarking method and data

We benchmarked 6 variant callers on datasets with spiked-in variants at low frequencies and the mBC dataset containing ctDNA. Four standard variant callers were first benchmarked on data without UMI sequences. Deduplication on non-UMI datasets was performed using GATK’s MarkDuplicates tool. We next benchmarked the 4 standard variant callers plus 2 UMI-aware variant callers on the same datasets with synthetic UMI sequences. Datasets with spiked-in variants were generated at approximately 200x, 450x and 850x read depths before deduplication (Table 2).

**Table 2:** Overview of NGS data used for benchmarking. Sequencing depths were mean values at all variant positions in spiked-in 850x, spiked-in 450x and spiked-in 200x datasets. Depths were calculated across target regions on mBC samples.

Sample Accession	Dataset	Panel	Original Depth	Post UMI Correction Depth	Post Mark-Duplicates Depth
*SRR10296599*	Spiked-in 850x	AVENIO ctDNA Expanded Kit	847	-	536
*SRR10296599*	Spiked-in 850x; synthetic UMIs	AVENIO ctDNA Expanded Kit	847	648	-
*SRR10296599*	Spiked-in 450x	AVENIO ctDNA Expanded Kit	449	-	298
*SRR10296599*	Spiked-in 450x; synthetic UMIs	AVENIO ctDNA Expanded Kit	449	334	-
*SRR10296599*	Spiked-in 200x	AVENIO ctDNA Expanded Kit	201	-	143
*SRR10296599*	Spiked-in 200x; synthetic UMIs	AVENIO ctDNA Expanded Kit	201	151	-
*SRR15081468*	mBC	custom 54-genes panel	765	-	129
*SRR15081468*	mBC; synthetic UMIs	custom 54-genes panel	765	166	-
*SRR15081470*	mBC	custom 54-genes panel	594	-	134
*SRR15081470*	mBC; synthetic UMIs	custom 54-genes panel	594	153	-
*SRR15081472*	mBC	custom 54-genes panel	605	-	231
*SRR15081472*	mBC; synthetic UMIs	custom 54-genes panel	605	239	-
*SRR15081477*	mBC	custom 54-genes panel	1023	-	164
*SRR15081477*	mBC; synthetic UMIs	custom 54-genes panel	1023	191	-
*SRR15081480*	mBC	custom 54-genes panel	826	-	245
*SRR15081480*	mBC; synthetic UMIs	custom 54-genes panel	826	273	-
*SRR15081482*	mBC	custom 54-genes panel	575	-	77
*SRR15081482*	mBC; synthetic UMIs	custom 54-genes panel	575	95	-
*SRR15081493*	mBC	custom 54-genes panel	740	-	232
*SRR15081493*	mBC; synthetic UMIs	custom 54-genes panel	740	266	-
*SRR15081494*	mBC	custom 54-genes panel	382	-	69
*SRR15081494*	mBC; synthetic UMIs	custom 54-genes panel	382	80	-

### Spiked-in datasets without synthetic UMIs: Mutect2 displayed the highest sensitivity at low frequencies

**Fig. 3:**
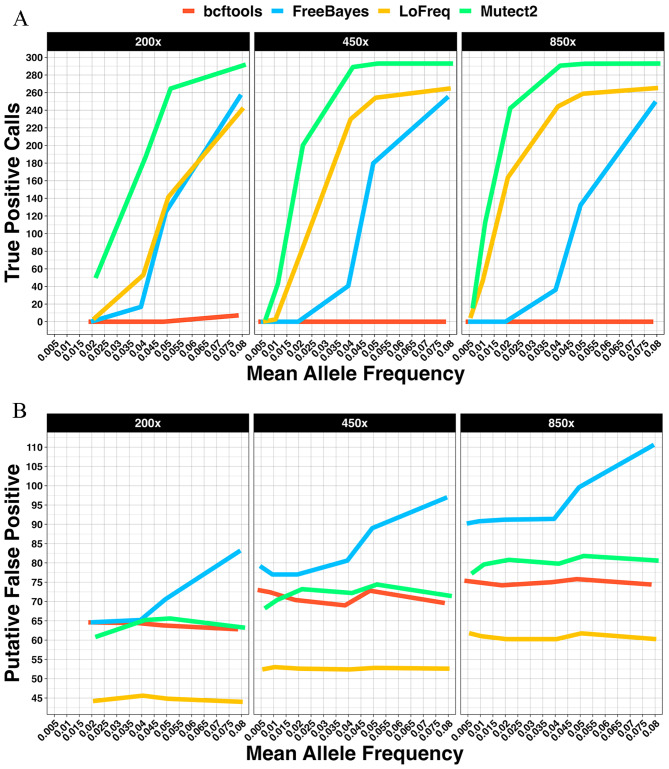
Mean true positive variants (**A**), and putative false positive variants (**B**) detected in datasets containing spiked-in variants without synthetic UMIs

Sensitivity across all variant callers increased with sequencing depth, as did discovery of putative false positive variants (Fig. 3A-B) in the datasets with spiked-in variants. Additionally, variant allele frequency increase returned an increase in the number of true positive variants detected.

Mutect2 detected a mean of 43 and 200 true positive variants at ∼0.01 and ∼0.2 VAF respectively in the spiked-in 450x dataset. Mutect2 also called more variants at higher VAF ranges tested, plateauing at 293 variants > = 0.06 VAF in the spiked-in 450x dataset. Bcftools only detected variants at ∼0.08 VAF at any depth.

In all three datasets containing spiked-in variants without synthetic UMIs, LoFreq detected the fewest putative false positive variants (Fig. 3B). The rate of putative false variants detected by FreeBayes increased with variant allele frequency, while remaining relatively stable for the other variant callers.

### mBC datasets without synthetic UMIs: variant callers displayed low concordance

**Fig. 4:**
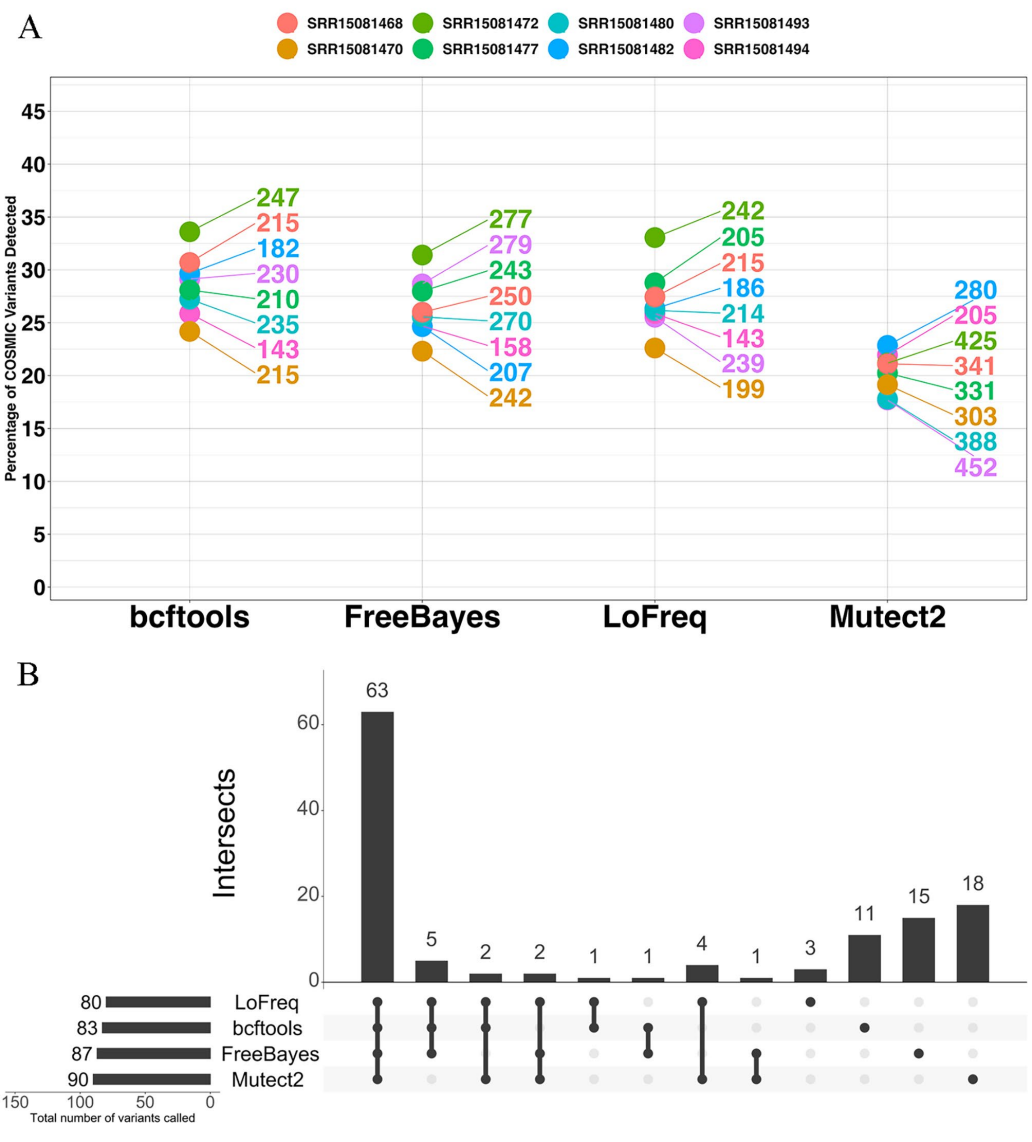
(**A**) Percentage of called variants in the mBC dataset reported in the COSMIC database. Labels indicate the total number of calls made. (**B**) Concordance of COSMIC variants in sample SRR15081472 from the mBC dataset. Horizontal bar plots indicate the number of COSMIC variants detected per caller. The vertical bar plot displays the number of intersecting variants by callers connected by points. Single point bars indicate the number of variants uniquely called by a variant caller

To gain an insight into the rate of true positive discovery in the absence of a truth set, we annotated filtered variants for presence in the COSMIC database. Mutect2 detected the lowest percentage of COSMIC variants across all 8 mBC samples, ranging between 17.7% and 22.9% (Fig. 4A). Bcftools detected the highest percentage of COSMIC variants ranging between 24.2% and 33.60%. Mutect2 generally called more variants than any other caller. For example, Mutect2 called 452 variants for sample SRR15081493, compared to 230, 279 and 239 detected by bcftools, FreeBayes and LoFreq respectively.

We investigated the concordance of COSMIC variants detected by callers across sample SRR15081472 to gain an insight into the rate of false positive variants detected. Concordance of variants detected across all 4 callers was 18.5% (Fig. 4B). LoFreq and Mutect2 had the highest concordance of any other individual pair of variant callers with 2.4% of variants called by only these 2 callers. Mutect2 called the largest fraction of privately called COSMIC variants at 20.0%. In comparison, bcftools, FreeBayes and LoFreq called 13.3%, 17.2% and 3.7% uniquely called COSMIC variants respectively.

### Spiked-in datasets with synthetic UMIs: UMI-VarCal detected the fewest putative false positive variants

**Fig. 5:**
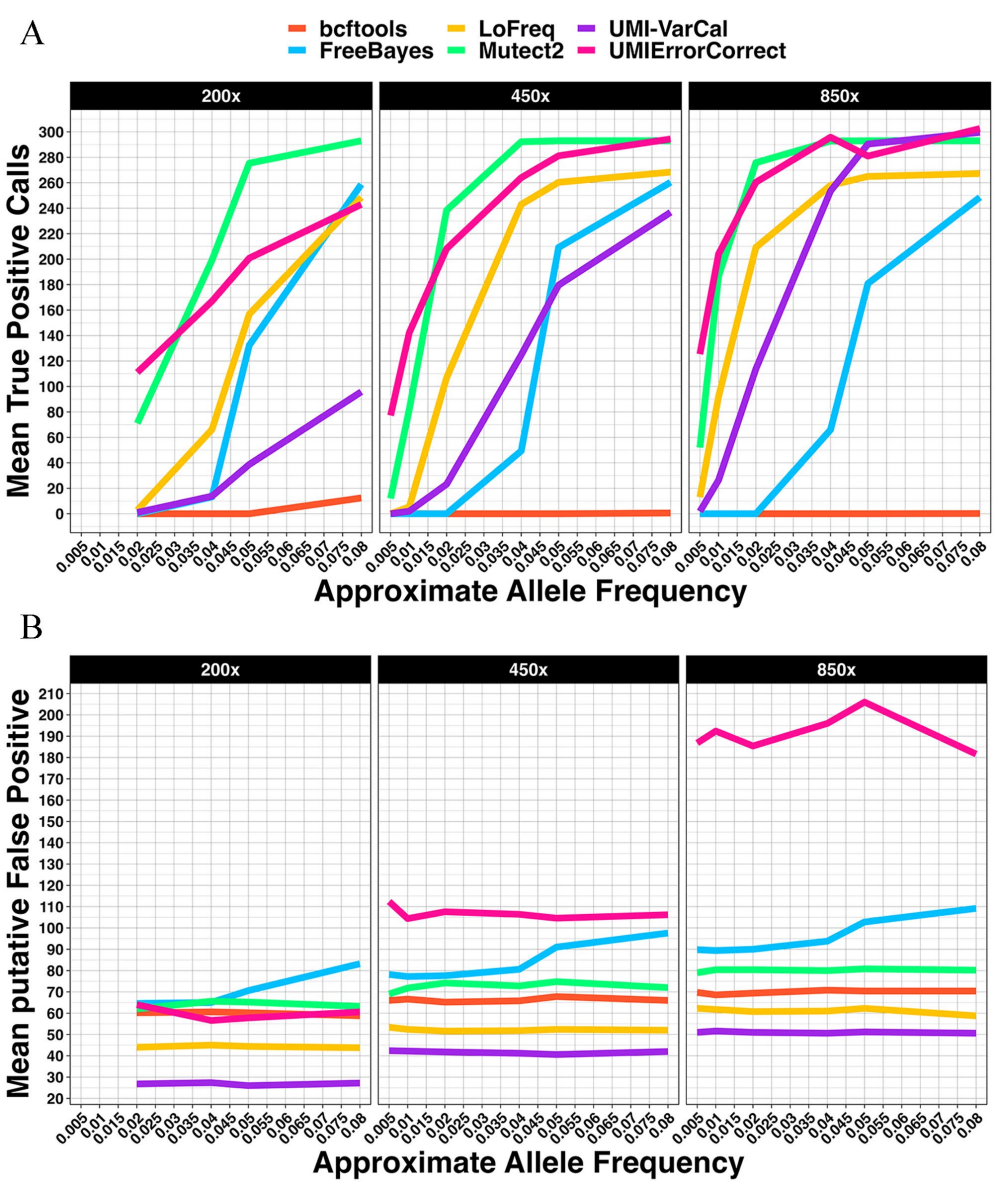
True positive calls (**A**) and putative false positive variants (**B**) detected at depths of 200x, 450x, and 850x in datasets with spiked-in variants and synthetic UMIs

We aimed to benchmark variant callers on NGS datasets that utilised UMIs for deduplication and error correction. Along with bcftools, FreeBayes, LoFreq and Mutect2, we also benchmarked two UMI-aware variant callers. UMIErrorCorrect detected more true positive variants than any other caller at the lowest VAFs investigated in all three datasets with spiked-in variants and synthetic UMIs (Fig. 5A). UMIErrorCorrect detected a mean of 110, 79 and 128 variants at the lowest VAFs in spiked-in 200x, 450x and 850x datasets respectively. The next most sensitive caller, Mutect2, detected a mean of 70, 11 and 52 variants in spiked-in 200x, 450 × 850x, with synthetic UMIs datasets respectively. As the depth increased, however, the number of putative false positives detected by UMIErrorCorrect increased substantially as shown in Fig. 5B. UMI-VarCal detected the fewest putative false positive variants across all variant callers while LoFreq called the fewest among standard variant callers.

### mBC data with synthetic UMIs: bcftools detected the highest percentage of COSMIC variants

**Fig. 6:**
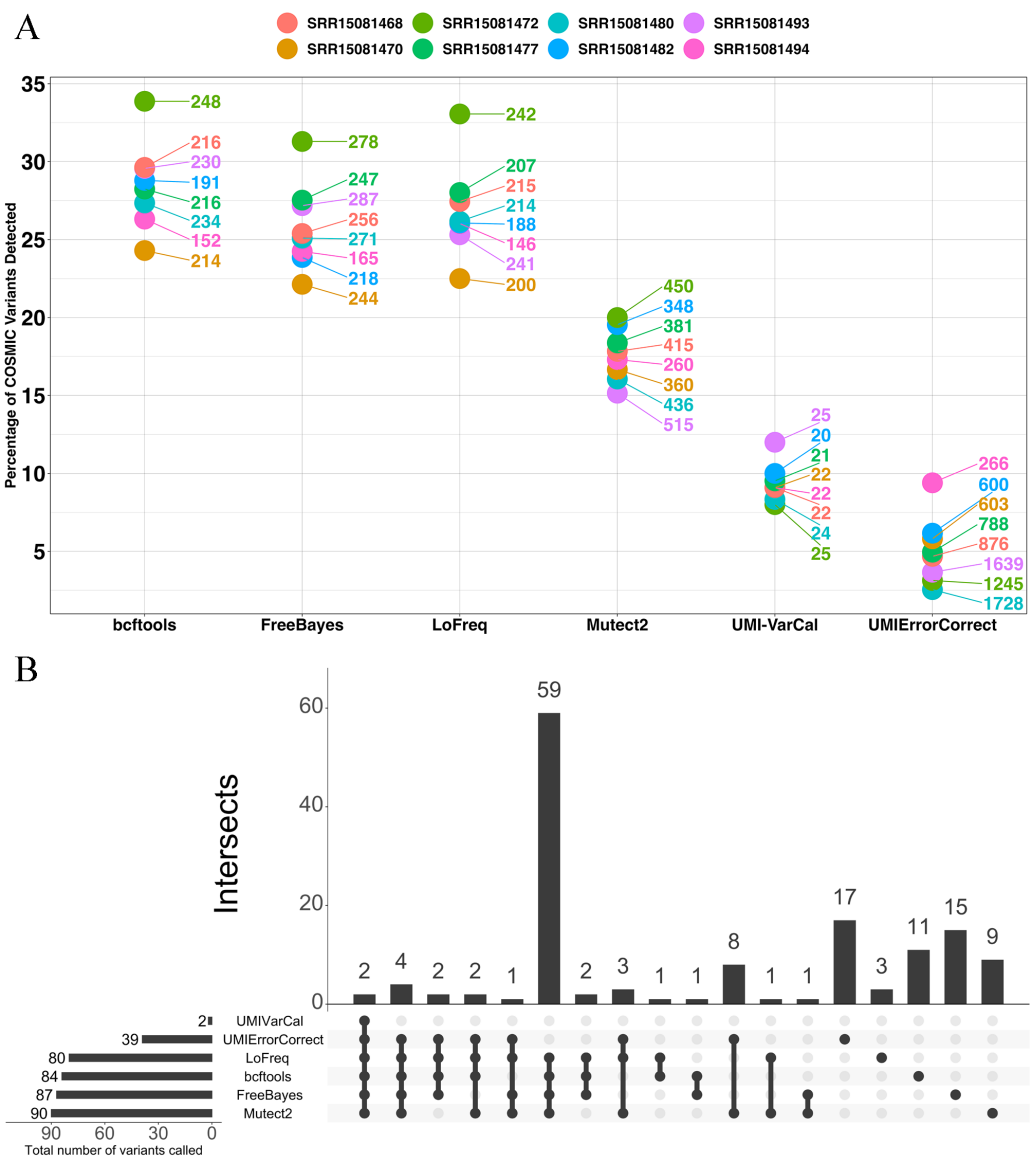
(**A**) Percentage of COSMIC variants detected by variant callers in data encoded with synthetic UMIs. Labels indicate the total number of variants detected. (**B**) Concordance of COSMIC variants in sample SRR15081472 from the mBC dataset with synthetic UMIs. Horizontal bar plots indicate the number of COSMIC variants detected per caller. The vertical bar plot displays the number of intersecting variants by callers connected by points. Single point bars indicate the number of variants uniquely called by a variant caller

In the mBC dataset with synthetic UMIs, bcftools detected a higher percentage of COSMIC variants than all other variant callers ranging between 24.3% and 33.9%. UMIErrorCorrect called the lowest fraction of COSMIC variants ranging between 2.5% and 9.4% (Fig. 6A). UMI-VarCal notably returned the lowest total number of variants. The caller detected 24 variants in sample SRR1501480, for example, while bcftools, FreeBayes, LoFreq, Mutect2 and UMIErrorCorrect detected 234, 271, 214, 436, and 1728 variants respectively. Figure 6B illustrates the COSMIC variant concordance in sample SRR1501472. The concordance of COSMIC variants across all 6 variant callers was 0.52%, while standard variant callers had a 17.3% concordance. Bcftools, FreeBayes, LoFreq, Mutect2, UMIErrorCorrect, and UMI-VarCal returned 13.1%, 17.2%, 3.8%, 10%, 43.6%, and 0% private COSMIC variants respectively.

There was no difference in the number of COSMIC variants detected by LoFreq, FreeBayes and Mutect2 between datasets with and without synthetic UMIs in sample SRR15081472. LoFreq, FreeBayes and Mutect2 detected 87, 80 and 90 COSMIC variants in both datasets respectively. Bcftools called one more COSMIC variant in the synthetic UMI datasets with 84 versus 83 COSMIC variants in datasets with and without synthetic UMIs respectively.

## Discussion

In this study we benchmark the performance of a set of UMI-aware and standard variant callers for discovery of variants in ctDNA data with and without synthetic UMIs. We made use of synthetic datasets as they allowed for the insertion of variants at user specified locations, depths, and frequencies, and the generation of UMIs which were otherwise unavailable. While there are bioinformatics tools for generating entirely synthetic short read synthetic data [39, 40], variant calling tools have been shown to overperform on these types of datasets as a result of the reduced complexity of output reads [41]. We therefore opted to spike in variants into a real cfDNA template sample. We additionally used a set of mBC samples from a clinical trial which will contain ctDNA.

Mutect2 called more variants at lower allele frequencies at all read depths in the spiked-in non-UMI datasets. In the mBC dataset, we used fraction of variants in the COSMIC database to infer true positive calls [42] with bcftools detecting more COSMIC variants than any other caller. It is worth noting using COSMIC for this exercise only provides an approximation of true positive variants detected; COSMIC is updated regularly with new somatic variants associated with cancer.

While Mutect2 detected a high number of true positive low frequency variants in spiked-in 200x, 450x, and 850x datasets, there were signs of high false positive discovery rate. In all three spiked-in datasets, Mutect2 called more putative false positive variants than LoFreq. The relatively high percentage of uniquely called variants and low percentage of COSMIC variants in the mBC samples also indicate low specificity from the caller.

Our results showed low concordance of COSMIC variants across all variant callers in sample SRR15081472 from the mBC dataset. Low concordance of variant calling tools has been reported in other studies [43, 44], which may be explained by differences in the internal algorithms used by variant calling tools and the challenging task of distinguishing between true positive ctDNA variants and artefacts. Concordance of variants can be used as an indicator of specificity in datasets where no truth set is available. Previous studies have shown commonly called variants are more likely to be true positive variants [45, 46]. In Bian et al. (2018)’s publication, an ensemble call set of FreeBayes, VarDict and Mutect consistently returned fewer false positive variants than any single variant caller benchmarked. Fang et al. (2015) used an ensemble call set of Mutect, VarScan2, SomaticSniper, JointSNVMix2 and VarDict as input into a machine learning classifier of high confidence somatic mutations.

Mutect2 borrows a local haplotype assembly algorithm from GATK’s germline variant caller, HaplotypeCaller [47]. When Mutect2 is processing a region with potential variation, reads mapping to the region are first locally reassembled before variant calling via a Bayesian somatic genotyping model. This strategy enables Mutect2 to detect difficult to call variants in complex regions. Indel calling sensitivity is particularly boosted by local reassembly of short sequence reads as these reads may not span an entire variant site making it difficult to characterise the variant. The preference for high sensitivity with Mutect2 comes with a trade-off in specificity as observed in our results.

Bcftools did not detect any variants at allele frequencies < ∼0.08 in any of the spiked-in datasets. As a general-purpose variant caller, bcftools was not written for low frequency variant detection. Other benchmarking studies have demonstrated bcftools struggles to detect variants below a variant allele frequency of approximately 0.2 [48]. Bcftools performed well on mBC samples where variant allele frequencies are expected to be higher than the values used in the spiked-in datasets. The caller detected a higher fraction of COSMIC variants than any of the standard variant callers in the samples both with and without synthetic UMIs. Bcftools also detected 13.1% unique COSMIC variants in data without synthetic UMIs, substantially fewer than Mutect2.

Overall, in the datasets without synthetic UMIs, LoFreq showed a balanced trade-off between sensitivity and specificity. In all datasets containing spiked-in variants, LoFreq detected the fewest putative false positive variants while only Mutect2 detected more spiked-in variants.

Unique molecular identifiers are increasingly being adopted for low frequency variant discovery in NGS experiments [33]. In this study we benchmarked the performance of 4 standard variant callers and 2 UMI-aware callers. Standard variant callers require a secondary tool to process UMI sequences before variant calling. We used fgbio for this task. UMI-aware variant callers, on the other hand, utilise internal algorithms to generate consensus reads. UMIErrorCorrect detected more variants at the lowest VAFs across all 3 read depths benchmarked, while UMI-VarCal consistently called the fewest putative false positive variants at all read depths benchmarked. The high sensitivity of UMIErrorCorrect and specificity of UMI-VarCal comes with a trade-off in specificity and sensitivity respectively. Mutect2 showed a good balance between sensitivity and specificity in the low frequency spiked-in datasets with synthetic UMIs.

Due to a lack of publicly available cfDNA data from cancer patients which also had publicly available UMIs, synthetic UMIs were generated and assigned to the data via a Poisson process. When low frequency variants were spiked-in to datasets, synthetic UMI families in which all reads contain the spiked-in variants were generated. However, these datasets may not reflect the true variation of ctDNA data. Synthetic UMIs were also added to the mBC dataset which contains ctDNA. Since the true ctDNA variants are unknown synthetic UMI families are not guaranteed to preserve them. Both these approaches combined enables us to benchmark the tools for comparison and to evaluate the utility of UMI-aware variant callers.

Collectively, the results of the benchmarking in synthetic UMI datasets show standard variant callers, utilising fgbio for collapsing UMIs, return call sets with a good balance between sensitivity and specificity. For calling low frequency variants, UMI-VarCal returned the fewest putative false positive variants and matched the high sensitivity of Mutect2 and UMIErrorCorrect in the spiked-in 850x dataset at allele frequencies over 5%.

### Limitations of the study

Best practice variant calling pipelines recommend base quality score recalibration (BQSR) of BAM files for somatic variant discovery [49]. This processing step involves recalibrating the base quality scores assigned by sequencing platforms when estimating the confidence in the bases called. As many variant calling algorithms rely on this metric to varying extents, an accurate quality score can be important. We did not perform this step in this benchmarking study because UMIErrorCorrect only accepts FASTQ files. The BQSR tools from GATK require BAM files as input and so to maintain a fair comparison, we opted not to recalibrate quality scores in the input BAM files for all other callers.

No depth or base quality score filters were applied to UMI-aware variant caller VCF files due to incompatibility with standard variant calling software. We used bcftools for variant filtering on depth and base quality thresholds – bcftools only accepts VCF files conforming to the VCF specification. While there was the option to filter UMI-aware VCF files manually, we concluded compatibility of output files with standard variant filtering tools is an important consideration for other researchers aiming to select the appropriate tools for their applications. We would encourage the development of tools with compatibility with other existing, widely used tools and the use of standard formats and naming conventions.

The variant filtering steps applied on standard variant caller VCF files, except Mutect2, included a filter to keep variant calls with 99.999% probability of being correct. While variant quality scores are assigned by the variant callers, and callers often report differing levels of confidence in the same variant, quality scores are a valuable filtering metric to help in filtering out artefacts. The lack of a quality score in Mutect2 VCF files may also be an indicator of why Mutect2 displayed signs of relatively low specificity on the mBC data set with and without synthetic UMIs. GATK provide external tools to filter Mutect2 calls, including the FilterMutectCalls tool. These tools have potential to increase the specificity of Mutect2, however, they are only compatible with Mutect2 VCF files. To maintain a fairer comparison of tools, we opted against using filtering tools designed for specific variant callers.

While the mBC data used in this benchmarking study was sequenced with UMI sequences, these were not included in the data made available for download from the SRA. As a result, we used a custom Python script to generate and assign synthetic UMIs to reads with the same start and stop positions – UMI families were determined by sampling from a Poisson distribution. Without UMI sequences, MarkDuplicates considers reads with the same start and end positions as duplicate reads as it’s not possible to determine if reads originated from multiple template molecules. UMIs enable more accurate deduplication in that reads with two different UMIs necessarily originated from different molecules even if they share the same start and end positions. A limitation of our approach is that it does not match the accuracy of real UMIs. This approach may result in genuine low frequency variants being filtered out where reads are erroneously assigned to UMI families. We would encourage the publicly available data sequenced with UMIs to also include the UMI sequences to enable these types of studies.

Finally, to ensure fair comparison, all variant callers were run with default parameters. Optimisation of parameters for each caller may have a significant impact on sensitivity and specificity. Mutect2 default parameters often result in calling of soft-clipped bases, likely increasing the false discovery rate of the caller. The aligner BWA-MEM was also run with default parameters, and optimisation may have an impact on the variants detected by callers as mapping quality is one of the metrics evaluated in calling candidate variants.

## Conclusions

Our results showed Mutect2 displayed high sensitivity with a trade-off in specificity on the mBC dataset with and without synthetic UMIs. At the low frequencies evaluated in spiked-in datasets, Mutect2 is more balanced in sensitivity and specificity. LoFreq showed balance between sensitivity and specificity while bcftools was not suited to low frequency variant calling.

The performance of UMI-VarCal and UMIErrorCorrect indicates utilising UMI sequences combined with UMI-aware variant callers can have a significant impact on sensitivity and specificity in ctDNA somatic variant discovery. Our results suggest, in some scenarios, internal processing of UMI sequences and variant detection can yield higher sensitivity and specificity than using third-party UMI processing tools like UMI-tools [50] and fgbio. To improve low frequency variant calling in ctDNA NGS data, there is a need for more UMI-aware variant calling tools.

We provide a reference to guide researchers on selecting tools for ctDNA somatic variant calling applications. In datasets with no UMI encoding, researchers may choose Mutect2 for variant calling where sensitivity is more important than minimising false positive variants, or LoFreq where a more balanced output is required. For NGS datasets with UMI sequences, researchers may opt to use UMI-VarCal which showed higher rates of specificity at low frequencies, or Mutect2 for a balance between sensitivity and specificity.

### Electronic supplementary material

Below is the link to the electronic supplementary material.

Supplementary Material 1

## Data Availability

Data supporting the findings of this study are publicly available on the Sequence Read Archive database. The synthetic data template is accessible under accession number SRR10296599 and at the following URL: https://www.ncbi.nlm.nih.gov/sra/?term=SRR10296599. Metastatic breast cancer samples are accessible under accessions SRR15081468, SRR15081470, SRR15081472, SRR15081477, SRR15081480, SRR15081482, SRR15081493, and SRR15081494 and at the following URL: https://www.ncbi.nlm.nih.gov/sra?linkname=bioproject_sra_all&from_uid=745047.

## Abbreviations

BAM: Binary Alignment Map
BED: Browser Extensible Data
cfDNA: Cell Free DNA
COSMIC: Catalogue of Somatic Mutations in Cancer
ctDNA: Circulating Tumour DNA
mBC: Metastatic Breast Cancer
NGS: Next Generation Sequencing
PCR: Polymerase Chain Reaction
SAM: Sequence Alignment Map
SRA: Sequence Read Archive
VAF: Variant Allele Frequency
VCF: Variant Call Format
UMI: Unique Molecular Identifier
